# Altered birefringence of peripapillary retinal nerve fiber layer in multiple sclerosis measured by polarization sensitive optical coherence tomography

**DOI:** 10.1186/s40662-018-0108-z

**Published:** 2018-06-17

**Authors:** Hong Jiang, Wan Chen, Silvia Delgado, Yi Liu, Ying Lin, Jianhua Wang

**Affiliations:** 1 Bascom Palmer Eye Institute, University of Miami Miller School of Medicine, 1638 NW 10th Avenue, McKnight Vision Research Building-Room 202A, Miami, FL 33136 USA; 2 Department of Neurology, University of Miami Miller School of Medicine, Miami, FL USA; 3 State Key Laboratory of Ophthalmology, Zhongshan Ophthalmic Center, Sun Yat-sen University, Guangzhou, China; 4 Department of Ophthalmology, Third Affiliated Hospital of Nanjing University of Chinese Medicine, Nanjing, China

**Keywords:** Remitting relapsing multiple sclerosis (RRMS), Peripapillary retinal nerve Fiber layer (pRNFL), Microtubule dysfunction, Polarization sensitive optical coherence tomography (PS-OCT)

## Abstract

**Background:**

The retina has been used to study the pathophysiology of multiple sclerosis (MS). Peripapillary retinal nerve fiber layer (pRNFL) thinning has been suggested as an ocular biomarker of neurodegeneration in MS. The goal of this project was to determine the birefringence of the pRNFL by measuring the fiber birefringence using polarization sensitive optical coherence tomography (PS-OCT).

**Methods:**

Sixty-six MS patients without history of optic neuritis (age: 39.9 ± 11.0 yrs. old, 53 females and 13 males) and 66 age- and gender-matched normal controls (age: 40.7 ± 11.4 yrs. old) were recruited. Custom built PS-OCT was used to measure phase retardation per unit depth (PR/UD, proportional to the birefringence) and pRNFL thickness in each quadrant of the pRNFL. In addition, clinical manifestation was used to correlate with the pRNFL birefringence.

**Results:**

The pRNFL was thinner in the temporal and inferior quadrants in MS patients compared with normal controls (*P* < 0.05). The PR/UD of the pRNFL was significantly decreased in MS patients (P < 0.05) in all quadrants except for the nasal quadrant. In both groups, the PR/UD from all four quadrants was not related to the averaged pRNFL thickness (*P* > 0.05). In MS patients, the PR/UD was not related to the expanded disability status scale (EDSS) nor disease duration (r ranged from − 0.17 to 0.02, *P* > 0.05).

**Conclusion:**

This is the first study using PS-OCT to study the pRNFL birefringence in MS patients. Decreased birefringence of the pRNFL may indicate microtubule abnormality, and could be a potential biomarker for detecting early neurodegeneration in MS.

## Background

Multiple sclerosis (MS) is an inflammatory demyelinating disorder in which axonal degeneration is known as the main pathological substrate underlying the progressive disability in patients with MS [1]. Visible MS lesions (focal demyelination associated with inflammation) shown on conventional magnetic resonance imaging (MRI) represent only a small fraction of MS pathology, whereas diffuse mild inflammatory degenerative alterations exist in normal appearing white and grey matters (NAWG) [2–4]. The non-conventional MRI, such as the diffusion tensor imaging (DTI) MRI, could quantify demyelization and axonal loss that is not visible on conventional MRI [5]. Similarly, the magnetization transfer (MTR) MRI can detect microstructural damage that is strongly correlated with the percentage of residual axons and the microscopic tissue damage in NAWG [4]. However, the DTI and MTR MRIs cannot detect axonal microstructural changes.

MS often involves the optic nerve presenting as acute optic neuritis or subclinical optic neuropathy and/or retrograde degeneration of the MS lesions within the posterior optic pathways [6]. The peripapillary retinal nerve fiber layer (pRNFL), composed of unmyelinated axons, has been suggested as a biomarker representing the axonal degeneration in the brain of MS patients. pRNFL can be noninvasively imaged by optical coherence tomography (OCT) [6]. However, monitoring the thickness of the pRNFL for detecting early microstructural changes of the axon may not be as sensitive. Instead, imaging its microstructural integrity may further improve the ability in detecting early axonal degeneration and monitoring the disease progression and treatment efficacy.

The pRNFL is known to exhibit birefringence [7], which is related to the structure of dominant axonal filaments, such as neurofilaments, axoplasmic membranes and microtubules [8]. Changes in the axonal cytoskeleton, such as neurofilament compactness and phosphorylation, resulting in related birefringence changes, usually precede axonal loss [9, 10]. Birefringence is a unitless difference of refractive indices and is expressed as delta *n*. Delta *n* is related to phase retardation/unit depth by the following equation: delta *n* = (λ/360) × (PR/UD), where λ is the central wavelength used in the measurement system, PR is the phase retardation, and UD is the unit depth [11]. We hypothesize that the pRNFL birefringence in MS patients is altered, which could be another indicator of axonal pathology in patients with MS. The pRNFL birefringence can be quantitatively measured using PS-OCT, which can assess the depth-resolved polarization properties of the tissue to provide additional information about tissue integrity [12, 13]. The goal of the present study was to determine the birefringence of the pRNFL in patients with relapsing-remitting MS (RRMS).

## Methods

**Fig. 1:**
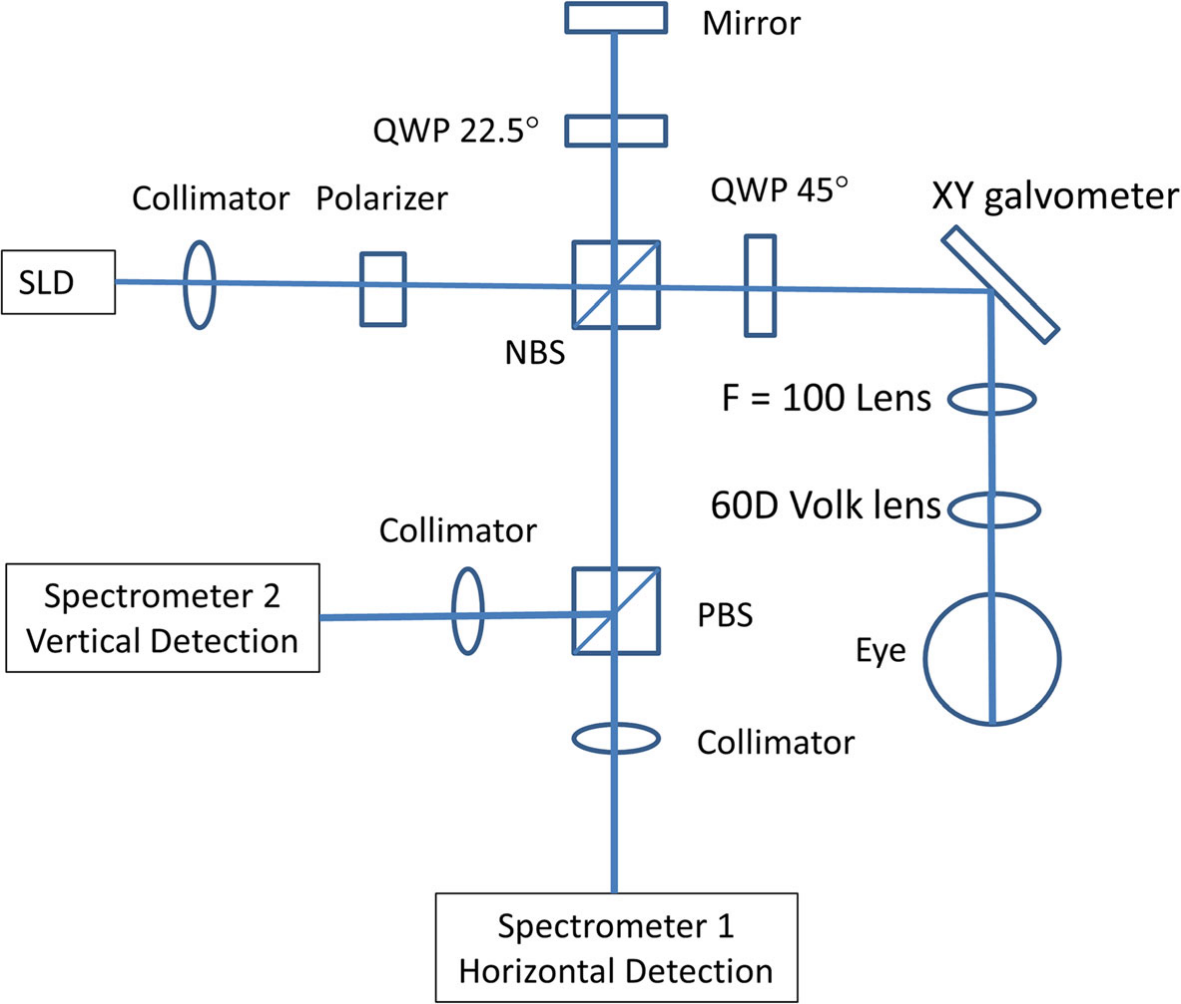
PS-OCT set up. SLD: superluminescent diode, QWP: quarter wave plate; PBS: polarizing beam splitter, NBS: non-polarizing beam splitter

The custom built PS-OCT was developed based on the configuration of spectral domain optical coherence tomography (OCT) with two identical spectrometers (Fig. 1) and a similar set up has been well described by others [14, 15]. In brief, a superluminescent diode laser-based light source (InPhenix Inc., Livermore, CA) with a center wavelength of 840 nm and a bandwidth of 50 nm is used, resulting in an axial resolution of ~ 6 μm in tissue. The light from the OCT light source enters a polarizer to generate vertically linear polarized light, which is split into two arms using a non-polarizing beam splitter. One arm is the sample arm to scan the retina and the other arm is the reference arm. Before the light enters the eye, it goes through a quarter wave plate (placed at 45 degrees). Similarly, the light in the reference arm goes through another quarter wave plate (placed at 22.5 degrees) before reaching a reference mirror. The returning light from both arms is separated using a polarizing beam splitter into vertical and horizontal detecting channels before reaching two identical spectrometers. The fringes of both vertical and horizontal reflected lights from the sample and reference mirror are detected and separated by these two spectrometers. Each of the spectrometers has a holographic diffraction grating (1200 lines/mm; Wasatch Photonics, Logan, UT) to span the fringe over 2048 sensor pixels on a line scan camera (Aviiva SM2 CL 2014, Atmel, San Jose, CA) with a projection lens (f = 180 mm).

**Fig. 2:**
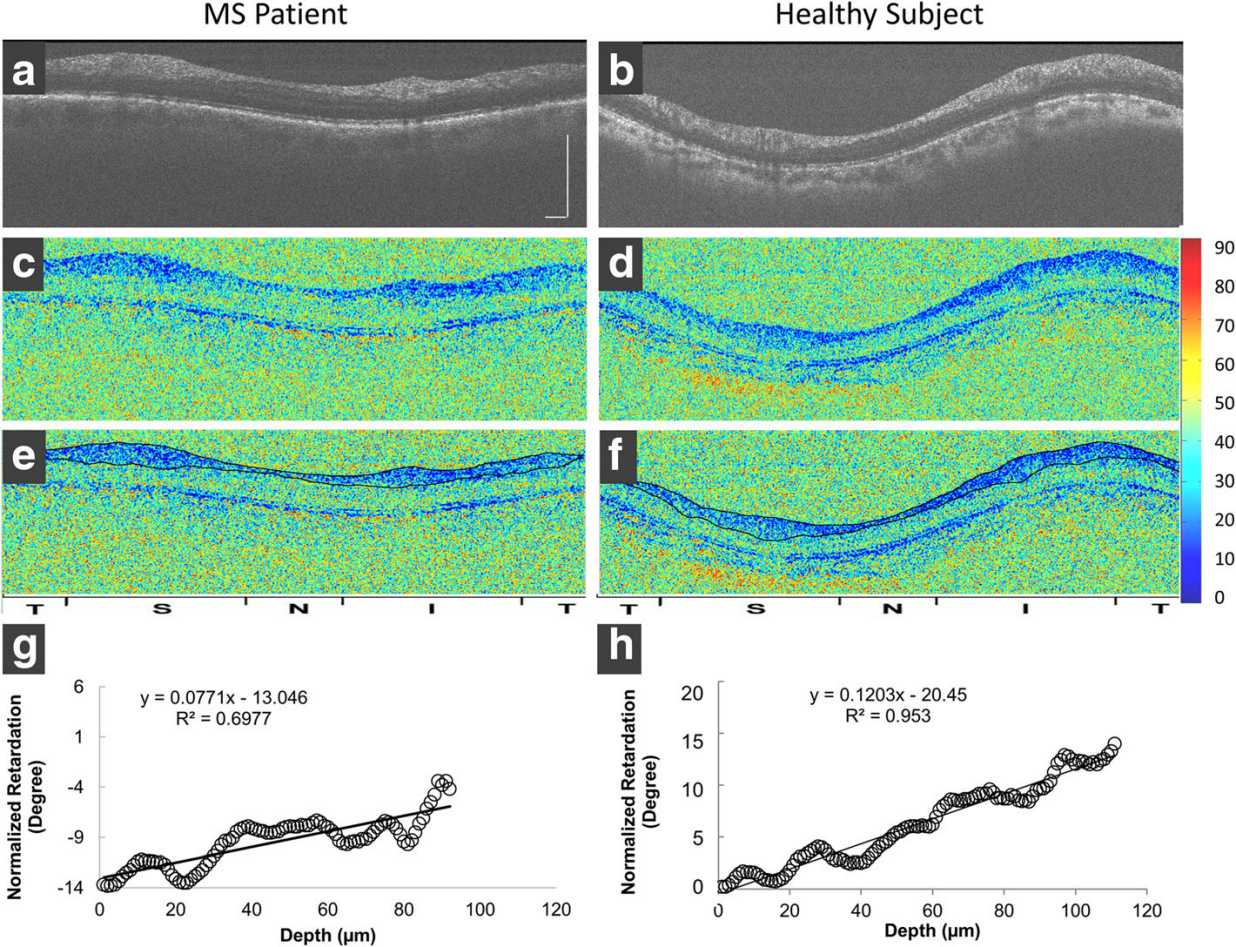
Polarization sensitive optical coherence tomography (PS-OCT) for imaging the pRNFL of a RRMS patient and healthy subject. Images of the pRNFL structure (**a** and **b**) and retardation (**c** and **d**) were obtained. The outlined pRNFL boundaries acquired from the OCT intensity images (**a** and **b**) were projected into the retardation images (**c**-**f**), creating segmented pRNFL with retardation (**e** and **f**). Averaged retardations of 512 A-scans from each quadrant were analyzed and plotted as a function of the scan depth in the RNFL. Scatter plots (**g** and **h**) were the averaged retardation in the inferior quadrant as a function of the scan depth. The slopes of the linear regression were defined as the PR/UD of the pRNFL. The PR/UD of the inferior pRNFL in the MS patient was 7.7 degrees/100 μm, which was lower than that in the control (12.0 degrees/100 μm). T = temporal; S = superior; N = nasal; and I = inferior. Bars = 500 μm

A circular scan with a diameter of 3.5 mm centered on the optic nerve head was scanned and datasets of both channels were processed using custom software developed in the MATLAB environment (Mathworks, Natick, MA). The program processes the acquired data for computing backscattered light reflectivity for imaging the tissue structure and phase retardation according to Jones matrix formalism [16]. Corneal birefringence changes the polarization state of the incident beam unpredictably [17]. While corneal birefringence compensation was applied in measuring retardation maps of the retina and pRNFL [15], the RNFL surface in the present study was used as a reference in the retardation calculation, so that corneal birefringence may not influence our measurement of PR/UD. The same method was used in a previous study [13]. The circle scan containing 2048 A-scans was partitioned by two meridians (45 and 135 degrees) into superior, inferior, nasal and temporal quadrants. Each quadrant contained a 512 A-scan. In the reflectivity image, the software detects the boundaries of the pRNFL. The background noise was calculated in the image area (10 × 10 pixels) where there was no tissue image and then subtracted from the entire image. Using the detected pRNFL layer information, the software extracts the retardation between the anterior and posterior boundary of the RNFL in each A-scan. The top pixel of the detected pRNFL of each A-scan was aligned and then the retardation of each pixel was averaged within the quadrant. The averaged retardation was then plotted against the depth (Fig. 2). For better plotting, signal normalization was done by setting the first pixel of the pRNFL to 0 in the x axis and the retardation in the first pixel to 0 in the y axis. This procedure did not alter the slopes obtained by the liner regression. Linear regression was performed and the slope was obtained since PR/UD is proportional to the birefringence value (delta *n*). Averaged birefringence values were calculated and used to represent the RNFL birefringence of the circular RNFL.

This study was approved by the institutional review board for human research at the University of Miami. Informed consent was obtained from each subject. All subjects were treated in accordance with the tenets of the Declaration of Helsinki. In this study, 66 RRMS patients (age: 39.9 ± 11.0 yrs. old, 53 females and 13 males) and 66 age- and gender-matched normal controls (age: 40.7 ± 11.4 yrs. old) were clinically assessed and recruited (Table 1). All RRMS patients were in the remitting stage. Their average expanded disability status scale (EDSS) was 1.8 ± 1.9. One eye of each subject was scanned. The scanned eye did not have a history of optic neuritis. Individuals with any other systemic, ocular or neurologic diseases were excluded. Subjects whose spherical and cylindrical refraction greater than + 6.0 or less than − 6.0 diopters were also excluded.

**Table 1:** Demographics and clinical manifestations of patients and normal subjects

	MS	Control
n	66	66
Age (yrs)	39.9 ± 11.0	40.7 ± 11.4
Gender	13 M 53F	13 M 53F
EDSS	1.8 ± 1.9	
DD (yrs)	6.4 ± 7.0	

Results are presented as the mean ± standard deviation.

Abbreviations: *M*: Male, *F*: Female, *MS*: multiple sclerosis, *EDSS*: expanded disability status scale, *DD*: disease duration

**Fig. 3:**
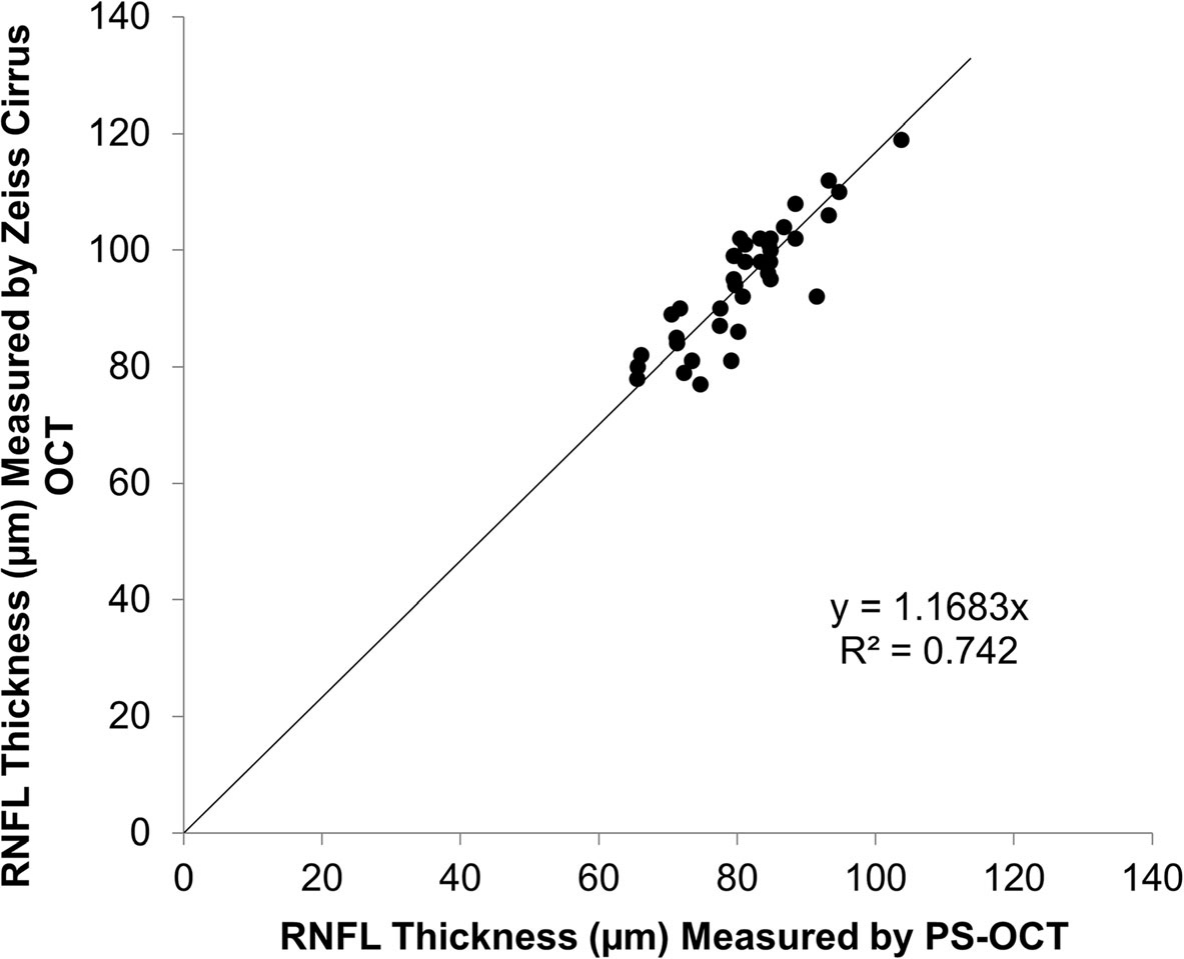
pRNFL thickness measured using Zeiss Cirrus OCT and PS-OCT. The pRNFL of a cohort of 38 subjects including 19 patients with RRMS was imaged using both Zeiss Cirrus OCT and PS-OCT.

For testing the reproducibility of the measurement using the PS-OCT system, measurements were taken twice on one eye of each of the 11 healthy subjects. The reproducibility (expressed as a percentage) was calculated as the standard deviation of the differences between two measurements divided by the mean. The coefficient of repeatability for the birefringence of the pRNFL was 13.0%. The measurement differences of pRNFL thicknesses using different OCT devices are reported, resulting in incompatibility of pRNFL measurements obtained using different systems by different manufacturers [18, 19]. To validate the pRNFL measurements using our custom PS-OCT device, 38 eyes of 38 subjects including 19 patients with RRMS were also imaged with the Zeiss Cirrus OCT (Carl Zeiss Meditec, Inc., Dublin, CA), and the results were compared with the measurements obtained from PS-OCT. The correlation of coefficient between the two systems was 0.87 (Fig. 3).

Descriptive statistics were used to summarize patient demographic and clinical information. All data were expressed as the mean ± SD. The thickness and the birefringence of the pRNFL between the two groups were analyzed by two-tail t-tests with two sample equal variance. Pearson’s correlation analysis was used to evaluate the strength of association between the thicknesses and the birefringence of the pRNFL. *P* values less than 0.05 were considered significant.

## Results

**Fig. 4:**
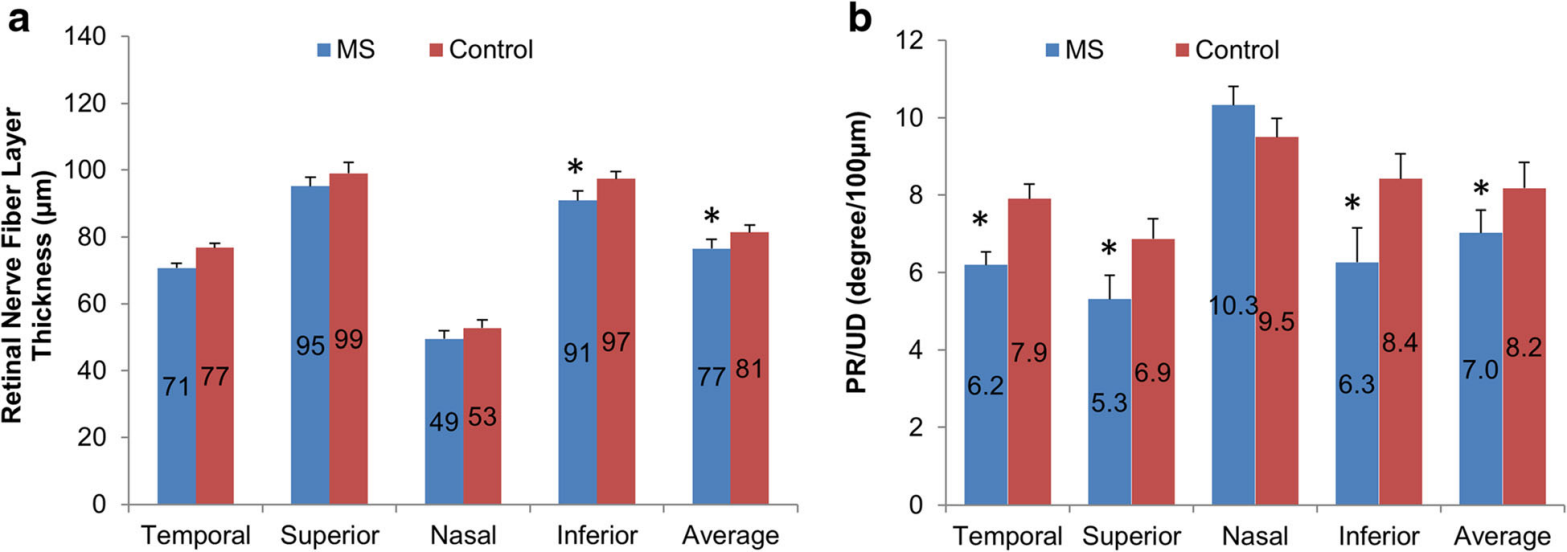
The thickness and birefringence of the pRNFL measured using PS-OCT. The thickness of the pRNFL in the inferior quadrant as well as the average thickness was thinner in MS patients compared with normal controls (**a**, *P* < 0.05). In addition, the PS-OCT derived PR/UD (proportional to birefringence) of the pRNFL was lower in all quadrants except for the nasal quadrant as well as the circular averaged PR/UD in patients with MS compared to age- and gender-matched normal controls (**b**, *P* < 0.05). *P < 0.05

The thickness of the pRNFL in the inferior quadrant as well as average thickness was thinner in MS patients compared with normal controls (*P* < 0.05, Fig. 4a). In addition, the PS-OCT derived PR/UD (proportional to birefringence) of the pRNFL was lower in all quadrants except for the nasal quadrant as well as the circular averaged PR/UD in patients with MS compared to age- and gender- matched normal controls (*P* < 0.05, Fig. 4b).

The averaged PR/UD was not correlated to averaged pRNFL thickness in both MS (*r* = − 0.10, P < 0.05) and control (*r* = − 0.02, P < 0.05) groups as well as in all subjects (*r* = − 0.001, P < 0.05). In MS, the average PR/UD was not related to EDSS and disease duration (r ranged from − 0.17 to 0.02, *P* > 0.05).

**Fig. 5:**
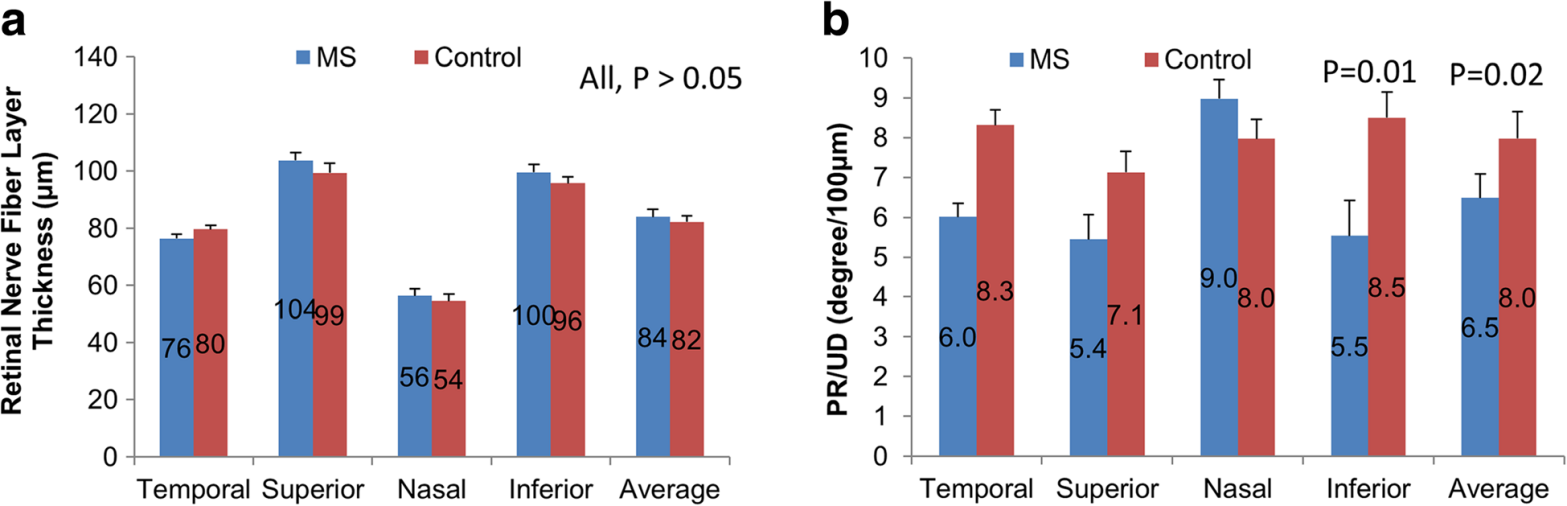
Peripapillary retinal nerve fiber layer (pRNFL) thickness and birefringence in MS patients. The MS patients with normal pRNFL thickness range formed a subject group using PS-OCT measured 78 μm as a cut off of pRNFL. These MS patients did not show a difference of pRNFL thickness compared to their paired controls (**a**, *P* > 0.05). In contrast, the PR/UD showed significant decreases in the inferior quadrant and the entire circle compared to their paired controls, respectively (**b**, *P* < 0.05)

To test whether the birefringence was changed in MS patients with no obvious pRNFL thinning (i.e. within normal range), the MS patients with normal pRNFL thickness range formed a subject group using PS-OCT measured 78 μm as a cut off of pRNFL. This cut off was about 90 μm using the conversion obtained in Fig. 3. The population mean of pRNFL is 100.1 ± 11.6 μm [20]. Therefore, these pRNFL thicknesses are more than the cut off value that can be regarded as the normal range. These MS patients did not show a difference of pRNFL thickness compared to their paired controls (Fig. 5, *P* > 0.05). In contrast, the PR/UD showed significant decreases in the inferior quadrant and the entire circle compared to their paired controls, respectively (*P* < 0.05).

## Discussion

In this study, MS patients were mildly disabled with relatively short disease duration. The eyes of RRMS patients in the remitting stage without a history of optic neuritis were studied, and the decreased birefringence found in the present study indicates the alteration of the microtubule structure abnormality in these patients. Furthermore, the alteration of birefringence appeared to exist in MS patients regardless of the pRNFL thickness. Birefringence alterations were not only found in MS patients with profound neurodegeneration as evident by pRNFL thinning, but also observed in MS patients whose pRNFL thickness were within normal ranges. Hence, pRNFL birefringence loss may precede pRNFL thinning, which could be an independent marker of early neurodegeneration. However, whether the change of birefringence occurs prior to pRNFL thinning may not be addressed in this cross-sectional study, and should be further investigated in future longitudinal studies.

In contrast to the PS-OCT with the depth resolved measurement of pRNFL birefringence, the scanning laser polarimetry (GDx) applies the polarized light on the retina to provide an integrated polarization measurement, so that the thickness of the retinal nerve fiber layer (RNFL) can be determined in MS patients [21, 22]. Many GDx studies have demonstrated that the pRNFL is thinner in MS patients as compared to healthy controls [21, 22]. However, the thinning of the pRNFL measured by GDx was not correlated with EDSS or other clinical parameters [23, 24]. Furthermore, GDx could not provide any in-depth information. Therefore, the birefringence (i.e. retardation per unit depth) cannot be measured because it measures the integrated phase retardation and then uses a fixed RNFL birefringence to convert phase retardation to RNFL thickness [25].

Unlike GDx, PS-OCT detects the polarization properties of the light collected from birefringent samples, such as the pRNFL [14, 26], which has been suggested to reflect the integrity of the pRNFL microstructure, such as microtubules [8]. It provides cross-sectional images of phase retardation, birefringence and optical axis information in the sample with depth information, using polarization-modulated light. Therefore, both structural information (traditional OCT structural image) and birefringence information (potentially connected to the integrity of retinal ganglion cell axon microtubules and neurofilaments) can be acquired simultaneously in two- and three-dimensions with a high resolution [26]. In other words, PS-OCT has the capacity to not only corroborate OCT findings of pRNFL thinning, but also provide insight into the microstructural damage that may precede or occur in the absence of pRNFL thinning identified by OCT, as shown in this study.

Our PR/UD measurements are slightly lower than the values of previous reports [27, 28]. Cense et al. reported that PR/UD in the healthy human retina ranged between 10 and 37 degrees/100 μm [13, 28]. The averaged PR/UD measurement in the present study was 8.2 degrees/100 μm in normal healthy subjects with a range between 2 and 17 degrees /100 μm. A similar range was found in the MS group although the average PR/UD was lower than controls. The discrepancy of the measurements in normal subjects may be due to different cohorts among studies or possible discrepancy of system calibration for scan depths. In addition, the difference of the numbers of A-scans used to plot the retardation as a function of tissue depth may also contribute to the slight difference of the measurements. We used 512 A-scans for the measurements and Cense et al. used fewer A-scans [13, 28]. While the measurement discrepancy warrants further investigation, this may not influence the comparison of the PR/UD between groups measured using the same device. There was a difference in the pRNFL thickness measurement between our custom PS-OCT and other OCT systems, such as the commercial Cirrus OCT. Our PS-OCT system was calibrated for its scan depth in air and converted from the optical distance to the geometric distance (by dividing tissue refractive index, 1.38 for the retina) and the slight difference we demonstrated in this study may be due to hardware configuration, segmentation algorithms and possible different scan location around the optic nerve head [18, 19]. However, a good correlation was found between our system and the commonly used Cirrus OCT.

Although a significant alteration of the pRNFL birefringence was found in MS patients for the first time, this study has some limitations. First, this was a cross-sectional study, which may not validate if RNFL thinning happened before the changes of birefringence. Second, the sample size may be a concern. The repeatability is about 13% and the change of the pRNFL birefringence was about 28%. To detect 20% of the change, a sample size of 21 subjects would have a detection power of 0.99, which was determined using a software program (GPower, Ver. 3.0) developed by Faul et al. [29]. Therefore, the sample size of 66 MS patients and matched controls was sufficient to detect the true change in birefringence.

## Conclusions

In summary, this is the first study using PS-OCT to study the pRNFL birefringence in MS patients. Decreased birefringence of the RNFL may indicate microtubule abnormality, which may be a potential biomarker for detecting early neurodegeneration in patients with MS.
